# Evidence for an effect of receptor density on ligand occupancy and agonist *EC*_50_

**DOI:** 10.1038/s41598-019-55361-x

**Published:** 2019-12-13

**Authors:** Gavin E. Jarvis, Andrew J. Thompson

**Affiliations:** 1 Department of Physiology, Development and Neuroscience, University of Cambridge, Cambridge, CB2 3EG UK; 2 Department of Pharmacology, University of Cambridge, Cambridge, CB2 1PD UK

**Keywords:** Neurophysiology, Ion channels in the nervous system

## Abstract

Drug-receptor interaction theory predicts that proportional receptor occupancy is a function of ligand concentration as defined by a ligand-receptor affinity constant, and is independent of receptor density. However, we previously observed that the *EC*_50_ of 5-HT reduced as the density of 5-HT_3_ receptors increased, suggesting an effect of receptor density on occupancy. The current study was designed to maximise variability in experimentally observed currents and confirm this apparent contradiction prospectively. *Xenopus* oocytes were injected with RNA encoding 5-HT_3_A receptors under conditions designed to achieve varying receptor expression levels and 5-HT-evoked currents measured using two electrode voltage clamp. Results from 99 oocytes showed that as the maximal peak current increased from 0.05 µA to 12.1 µA there was a 3.7-fold reduction in *EC*_50_. Since occupancy and conductance are directly related in this system, this indicates that for a given concentration of 5-HT, proportional occupancy increases with increased receptor density. We conclude that normalising data masks this correlation, and can result in reduced accuracy of pharmacological measurements. We propose a mechanistic explanation for our observations.

## Introduction

Receptor-mediated responses are non-linear functions of agonist concentration and the relationship between drug concentration and receptor occupancy is often modelled using the Hill-Langmuir equation^1,2^. When the intrinsic drug-receptor interaction remains unchanged and the drug is in relative excess, proportional receptor occupancy is predicted to be constant for a particular drug concentration. For many ligand-gated ion channels, there is a close and direct relationship between this receptor occupancy and the current response. In the absence of agonist, most channels remain closed and there is no observable current. However, when agonist binds the open probability of the channels increases and currents are observed. Thus, the concentration of agonist at which 50% of the ligand-gated ion channels are occupied (i.e., the dissociation constant, *K*_*d*_) would be the same as the *EC*_50_ (i.e., the concentration at which 50% of the maximal current is achieved). Given a model in which proportional occupancy is constant for a given ligand concentration, *K*_*d*_ and *EC*_50_ would be expected to remain the same regardless of channel density.

Since the magnitude of whole cell currents is dependent on channel expression levels, measured currents are frequently normalised to a specified (often maximally observed) value to eliminate substantial variance in response that arises because of these varying levels of channel expression. Such normalisation implies that *K*_*d*_ and *EC*_50_ are independent of channel density and eliminates the possibility of recognising covariance between maximal current and other pharmacological parameters that define the response. Consequently, normalisation may introduce bias into estimates of these other parameters.

Previously, we used non-linear mixed effects modelling to analyse non-normalised current data to investigate the effects of terpenoids on 5-HT_3_ receptors. We noted an unexpected correlation between the maximal peak current response (*I*_*max*_) and agonist *EC*_50_^3^. Since 5-HT_3_ receptors have a unitary conductance^4–7^, and a fundamental determinant of *I*_*max*_ is the number of cell-surface receptors, this finding suggested that as receptor expression increased, ligand occupancy (and therefore sensitivity to 5-HT) also increased, contrary to the theoretical expectations outlined above. 5-HT_3_ receptors are ligand-gated ion channels that contain an integral ion channel and they are therefore an ideal model for studying the relationships between agonist concentration, receptor expression and proportional occupancy, as RNA and DNA encoding them can readily be introduced into cells, and their expression on the cell-surface makes them amenable to electrophysiological measurements. When 5-HT binds at extracellular sites, it opens a transmembrane pore that allows ions to flow across the cell-surface membrane with unitary conductance. Thus, the relationship between occupancy and functional response is direct and proportional, unlike other receptor systems, such as G-protein-coupled receptors.

Explicit recognition that ion channel density affects agonist sensitivity would refine our understanding of pharmacological action. Therefore, in this prospective study, we adjusted experimental conditions to generate oocytes with a wide range of 5-HT_3_ expression levels and resultant 5-HT current responses. We confirm that *EC*_50_ and *I*_*max*_ are correlated, explore potential mechanistic explanations, and consider the implications for pharmacological measurements.

## Results

### 5-HT-evoked currents in Xenopus oocytes

5-HT-evoked currents were recorded at −60 mV from 99 injected oocytes. These oocytes had been injected with varying doses of RNA and incubated for varying time periods before recording the 5-HT response. Of these, 4 had no response to high concentrations of 5-HT (3–10 µM) and therefore only 1 or 2 recordings were made in each case. In these 4 oocytes, the dose of injected RNA was between 1.58 and 14.35 ng and the mean time between injection and recording was 5.2 hr (standard deviation = 0.3 hr) (Supplementary Table 1). For the remaining 95 oocytes, the number of recordings per oocyte was chosen to enable modelling of full concentration-response curves. Of these, 80 had 7.1 ± 0.7 (mean ± SD) recorded 5-HT applications per oocyte with none having fewer than 5, and 15 oocytes had either 14 or 15 recordings each (Supplementary Table 1). The 5-HT concentration-response relationships for these 95 oocytes were modelled and a summary of these results is presented in Table 1.

**Table 1:** Summary results of modelled 5-HT concentration-response relationships. Results are from 95 oocytes that responded to 5-HT. Data were fitted such that 0 ≤ *γ* ≤ 2.

Parameter	N	Mean	SD	Minimum value	Maximum value	Skew	Kurtosis
*I*_*max*_ (-µA)	95	5.26	3.35	0.050	12.1	−0.079	−1.09
*pEC*_50_	95	5.78	0.17	5.44	6.16	0.48	−0.40
*n*_*H*_	95	3.94	0.49	2.74	5.89	1.44	3.47
*α*	74	0.072	0.064	0.0063	0.29	1.77	2.74
*log*_10_ *α*	74	−1.30	0.37	−2.20	−0.53	−0.15	0.01
*γ*	74	0.99	0.28	0.26	1.68	−0.09	−0.05

In 18 cases, *γ* was constrained to 2, and in 3 cases to 0. Summary values for *α*, log_10_ *α* (*α* was log-normally distributed) and *γ* are from the 74 cases that were unconstrained. (*α* and *γ* are defined in Materials and Methods).

Positive values of *γ* (Table 1) indicate that the residual error is heteroscedastic. Therefore, parameter estimates are expected to be more reliable using an extended least squares (*ELS*) objective function, as compared to those derived with the more commonly used ordinary least squares (*OLS*) method, which assumes homoscedastic error variance. A good example of this was the estimate of the Hill coefficient: *n*_*H*_ = 4.21 ± 3.27 (skew = 5.4; kurtosis = 30.0; n = 95) when modelled using *OLS*, indicating reduced accuracy and precision in the estimate of this parameter.

**Figure 1:**
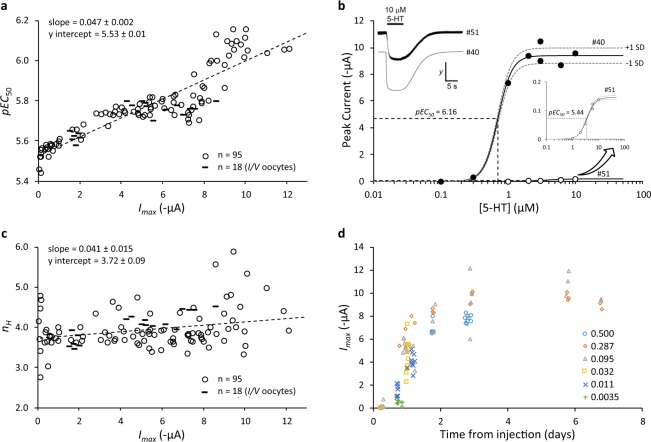
Concentration-dependency of 5-HT-evoked currents in *Xenopus* oocytes expressing human 5-HT3A receptors. (**a**) A plot of *I*_*max*_ vs *pEC*_50_ values obtained from 95 oocytes (○) in which a 5-HT response was detected. A linear regression model (- - - -) and parameters (mean ± SE) are shown, and the correlation coefficient [99% CI], *r* = 0.91 [0.84, 0.94] (calculated using GraphPad Prism 8.3.0). (**b**) Illustrative 5-HT concentration-response relationships from two oocytes, one with the highest (●, #40) and one with the lowest (○, #51) *pEC*_50_ values recorded in our study. The insert on the lower right is a magnification of the concentration-response curve from oocyte #51. Dashed lines indicate the *pEC*_50_ values and ± 1 standard deviation around the fitted model as defined by *α* and *γ* parameters, reflecting the increase in error variance as the response increases. The insert on the upper left shows current traces from these oocytes with 10 µM 5-HT. The *y* scale bar represents 0.1 µA for oocyte #51 and 5 µA for oocyte #40. Trace images were generated using Strathclyde Electrophysiology Software Package v4.7.3 (http://spider.science.strath.ac.uk/sipbs/software_ses.htm; University of Strathclyde, UK). (**c**) A plot of *I*_*max*_ vs *n*_*H*_ values obtained from the same 95 oocytes as Panel (a). The correlation coefficient [99% CI], *r* = 0.28 [0.02, 0.50] (calculated using GraphPad Prism 8.3.0). (**d**) A plot of *I*_*max*_ vs time from injection for a range of injected RNA concentrations. Concentrations of injected RNA (µg·µl^-1^) are as follows: ○ 0.500; ⬦ 0.287; △ 0.095; **☐** 0.032; × 0.011; + 0.0035. (Additional data in Panels (a) & (c) are an independent set of 18 replicates (–) in which full current-voltage (*I/V*) analysis was also performed. These data show the parameter values at −60 mV).

Figure 1a is a plot of *I*_*max*_ vs *pEC*_50_ for all 95 oocytes. For the full range of observed maximal peak currents (0.05–12.1 µA), there was a strong correlation between *I*_*max*_ and *pEC*_50_ (Pearson *r* = 0.91 [99% confidence interval: 0.84, 0.94]) with a predicted 3.7-fold difference in the agonist *EC*_50_ as indicated by linear regression. Illustrative concentration-response curves for the oocytes with the highest (oocyte #40: *I*_*max*_ = 9.4, *pEC*_50_ = 6.16, *n*_*H*_ = 4.17) and lowest (oocyte #51: *I*_*max*_ = 0.15, *pEC*_50_ = 5.44, *n*_*H*_ = 2.74) *pEC*_50_ values are in Fig. 1b. 5-HT_3_ channels have a unitary conductance, and therefore *I*_*max*_ is a function of the number of receptors on the cell-surface. Hence, the observed correlation suggests that as receptor density increases, so does agonist sensitivity. By contrast, the correlation between *I*_*max*_ and *n*_*H*_ was weak (Pearson *r* = 0.28 [99% confidence interval: 0.02, 0.50], Fig. 1c). *I*_*max*_ increased with time following RNA injection. Most of this increase occurred within the first 2 days post injection, after which there was little change in the observed maximal peak current (Fig. 1d).

### pEC_50_ and n_H_ are independent of current amplitude

**Figure 2:**
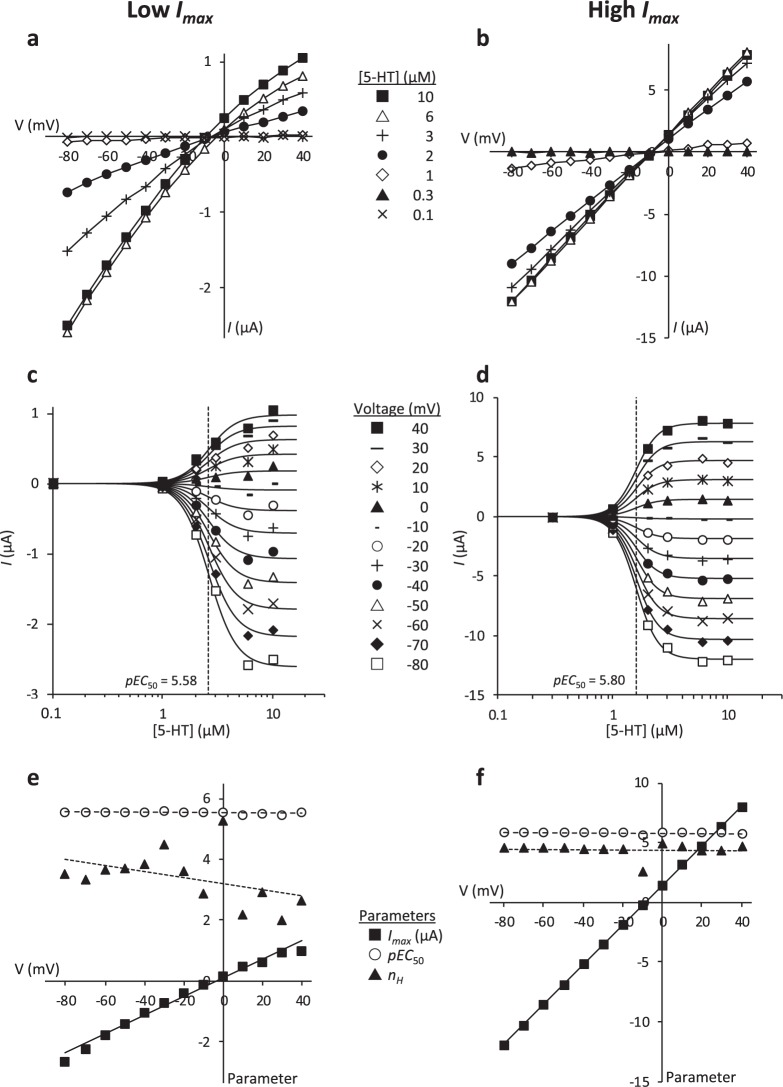
The effect of holding potential on peak current response and *pEC*_50_ values. Illustrative current-voltage (*I/V*) data from two different oocytes that responded with low (**a**,**c**,**e**) and high (**b**,**d**,**f**) maximal peak current responses. (**a**,**b**) Current-voltage (*I/V*) plots at different 5-HT concentrations (■ 10 µM; △ 6 µM; + 3 µM; ● 2 µM; ⬦ 1 µM; ▲ 0.3 µM; × 0.1 µM). (**c**, **d**) Peak current shown as a function of 5-HT concentration at differing holding potentials (−80 to + 40 mV in 10 mV steps). The fitted curves represent the Partial Model in which *pEC*_50_ and *n*_*H*_ parameters are constrained to be the same across all holding potentials for each oocyte. The *pEC*_50_ is indicated by the dotted line. (**e**, **f**) The relationship between the fitted parameters, *I*_*max*_, *pEC*_50_ and *n*_*H*_, and the holding potential. The values shown are from the Full Model in which the parameters can adopt different values for each holding potential. The figures illustrate the variance in the unconstrained estimates of *pEC*_50_ and *n*_*H*_. Likelihood Ratio Tests comparing the Full and Partial Models suggest that there is no difference in the *pEC*_50_ and *n*_*H*_ values between different holding potentials (Low responder: *P* = 0.74; High responder: *P* = 0.93). The straight line gradients (mean ± SE) of the data in Panel (e) are: *I*_*max*_, 0.031 ± 0.001 (*P* = 3 × 10^-11^); *pEC*_50_, −0.00051 ± 0.00025 (*P* = 0.063); *n*_*H*_, −0.0100 ± 0.0062 (*P* = 0.14), and in Panel (f) are: *I*_*max*_, 0.17 ± 0.0004 (*P* = 5 × 10^-24^); *pEC*_50_, −0.00015 ± 0.00029 (*P* = 0.62); *n*_*H*_, −0.0013 ± 0.0045 (*P* = 0.78). Note the differing *y* axis scales in the panels.

To ensure that the observed change in *pEC*_50_ was not an artefact of the current amplitude, we also measured 5-HT-evoked responses at 13 different holding potentials (−80 to + 40 mV, in 10 mV steps) in 18 additional oocytes. By varying clamp voltage receptor density remained the same, but peak current varied with holding potential. Figure 2 shows example data from oocytes with high and low *I*_*max*_ values. To evaluate whether *pEC*_50_ and *n*_*H*_ were independent of holding potential, data from each oocyte were modelled to: (1) a Full Model comprising 13 concentration-response curves with individual *I*_*max*_, *pEC*_50_ and *n*_*H*_ parameters for each voltage; and (2) a Partial Model with *pEC*_50_ and *n*_*H*_ parameters constrained across all voltages and 13 individual *I*_*max*_ values. *α* and *γ* were constrained to be equal for all holding potentials within both models. For each oocyte, the Full and Partial Models were compared using a Likelihood Ratio Test (degrees of freedom = 24) giving *P* values ranging from 0.0004 to 1.000 (median [interquartile range] = 0.83 [0.20, 0.99]). The gradients of holding potential vs *pEC*_50_ and *n*_*H*_ were calculated for each oocyte using the parameter estimates from the Full Model. For the oocytes with low *I*_*max*_, the gradient ( ± standard error) for the *pEC*_50_ values was −0.00051 ± 0.00025 (Fig. 2e). For oocytes with high *I*_*max*_, it was −0.00015 ± 0.00029 (Fig. 2f). For all 18 oocytes, the mean gradients [95% confidence intervals] were: −0.00006 [−0.00020, 0.00009] for *pEC*_50_, and 0.0010 [−0.0020, 0.0040] for *n*_*H*_. Taken together, these results indicate that *pEC*_50_ and *n*_*H*_ were unaffected by the holding potential or the associated change in the peak current. The Partial Model estimates of *pEC*_50_ and *I*_*max*_ recorded at −60 mV in these 18 oocytes matched those from the larger (n = 95) data set (these data are included for comparison in Fig. 1a). The results from these 18 oocytes are therefore a further independent replication of our primary experiment.

## Discussion

The data in this study show that peak *I*_*max*_ and *pEC*_50_ of 5-HT_3_ receptors are correlated. The assumption that peak *I*_*max*_ is a good measure of receptor expression is logically well-founded since 5-HT_3_ receptors have a unitary conductance^4–7^, and their desensitisation is minimal in the experimental system described here^3,8–10^ (Fig. 1b, *insert*). By using electrophysiology to quantify receptor expression, individual estimates of *I*_*max*_ and *pEC*_50_ are obtained from data collected under the same conditions. This within-subject design eliminates variance that would be introduced by measuring receptor expression using alternative methods such as radioligand binding, immunohistochemistry or Western blotting. Nevertheless, radioligand binding studies on GABA_c_ receptor have previously confirmed a correlation between current measurements and channel expression^11^, providing further evidence that *I*_*max*_ is proportional to the total number of expressed cell-surface receptors. Given that receptor activation and therefore conductance are dependent on binding of 5-HT, these results suggest that receptor occupancy and consequently the *pEC*_50_ are partially dependent upon the cell-surface density of the receptors.

**Figure 3:**
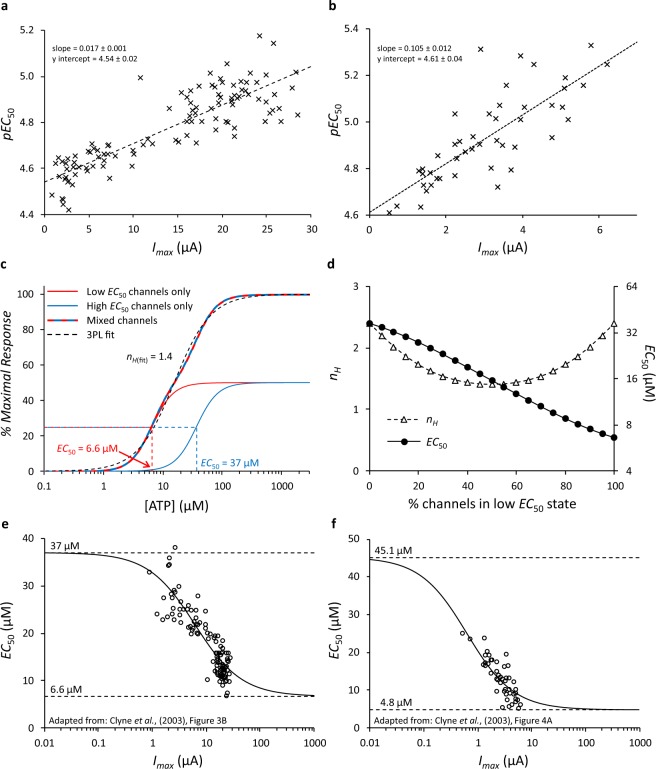
A representation and review of P2X2 data from Clyne *et al*. (2003). *I*_*max*_ and *pEC*_50_ from (**a**) native P2X2 receptors shown in Fig. 3B of Clyne *et al*.^12^ and (**b**) C-terminal 76 amino acid deleted P2X2 receptors shown in Fig. 4A of Clyne *et al*.^12^. Values were estimated by visual inspection and represented in a similar fashion to Fig. 1a in this report. (**c**) A simulation of the predicted response (fitted to the 3PL equation) resulting from two distinct populations of channels each with similar expression levels, Hill coefficients of 2.4, but with different *EC*_50_ values equal to 6.6 µM (red) and 37 µM (blue). The mixed population of channels has an intermediate *EC*_50_ of 15.6 µM and an apparently lower Hill coefficient of approximately 1.4. (**d**) Predicted change in apparent Hill coefficient (*n*_*H*_) and *EC*_50_ for a mixed population of the two channel subtypes defined in Panel (**c**), as the proportion of those subtypes changes from 0 to 100%. (**e**) A representation of Fig. 3B from Clyne *et al*.^12^ with the *x* axis shown on a log scale. The line is the hyperbolic model fitted by Clyne *et al*.^12^ indicating a high *EC*_50_ of 37 µM, a low *EC*_50_ of 6.6 µM and a mid-point *I*_*max*_ of 6.108 µA, as described in their manuscript. (**f**) An equivalent representation of Fig. 4A from Clyne *et al*.^12^. The line is the fitted hyperbolic model indicating a high *EC*_50_ of 45.1 µM, a low *EC*_50_ of 4.8 µM and a mid-point *I*_*max*_ of 0.637 µA. Panels (**e,f**) illustrate how the parameter estimates lie in relation to the data. Note that Clyne *et al*.^12^ constrained the lower *EC*_50_ values to be no smaller than the lowest measured *EC*_50_ and consequently the low *EC*_50_ limits (dashed lines) pass through the lowest value data points.

**Figure 4:**
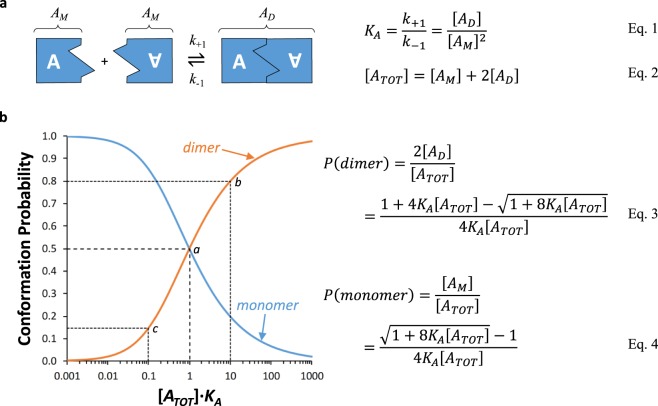
Modelling the formation of dimers from individual channels, A. (**a**) The dimerisation model assumes mass action association and dissociation of A channels. [*A*_*M*_] is the concentration of A monomers, and [*A*_*D*_] the concentration of dimers formed from two A channels. The association constant (*K*_*A*_) is the ratio of the forward (*k*_+1_) and backward (*k*_−1_) rate constants and is determined by the concentrations of *A*_*D*_ and *A*_*M*_ at equilibrium (Eq. 1 in Figure). [*A*_*TOT*_] is the concentration of all A channels whether they form a monomer or dimer (Eq. 2). (**b**) The model predicts that, as [*A*_*TOT*_] increases, the probability that the channels form dimers also increases. Thus, when [*A*_*TOT*_] = the dissociation constant (*K*_*d*_ = 1/*K*_*A*_), the proportion of A channels forming either a monomer or a dimer is 0.5 (‘*a*’ on figure). When [*A*_*TOT*_] is high, channels are therefore more likely to form dimers. When [*A*_*TOT*_]·*K*_*A*_ = 10 (‘*b*’), the proportion of A channels forming dimers is 0.80, whereas when [*A*_*TOT*_]·*K*_*A*_ = 0.1 (‘*c*’), the proportion is 0.15. Models describing the probabilities of dimer (Eq. 3) and monomer (Eq. 4) formation clearly reveal that these are dependent on [*A*_*TOT*_].

A similar relationship between *I*_*max*_ and *EC*_50_ has been reported for ATP acting at P2X2 receptors expressed in *Xenopus* oocytes^12,13^. Clyne *et al*.^12^ also proposed two mechanisms to account for their observations. We therefore examined Clyne’s data in more detail, estimating values of *I*_*max*_ and *pEC*_50_ from their Fig. 3B (native P2X2 receptors) and Fig. 4A (mutant P2X2 receptors). Using the same graphical representation that we used for our results, Clyne’s data are shown in our Fig. 3a,b. The similarity with our data in Fig. 1a is clear.

Clyne *et al*.^12^ proposed two mechanistic models. Firstly, they suggested that P2X2 receptors might bind to an endogenous membrane protein present at a fixed concentration that causes bound receptors to have a higher *EC*_50_. They suggested that at higher P2X2 receptor densities these endogenous binding proteins would become saturated resulting in proportionally more P2X2 receptors being unbound, thereby manifesting a lower *EC*_50_ overall. Secondly, they suggested that dimerised channels might have a lower *EC*_50_ compared to monomers and that increased expression would increase the proportion of channels in a dimeric conformation. High expression would therefore cause a reduction in the overall *EC*_50_. Indeed, the occurrence of multimers is not without precedent as P2X1 receptor clustering is seen in the *Xenopus* expression system^14^.

However, Clyne’s models do not fully account for the observed data, as a mixed population consisting of two functionally distinct receptor subtypes with differing *EC*_50_ values for the same agonist will manifest as a receptor population with an intermediate *EC*_50_ value and varying effects on the concentration-response shape and Hill coefficient. When the difference in the *EC*_50_s is large, a mixed population will appear as a biphasic concentration-response relationship (similar to that found by Taleb & Betz^15^ at glycine receptors). When the difference in *EC*_50_s is small, the outcome will appear monophasic with a shallower Hill coefficient (*n*_*H*_) than either of the distinct receptor subtypes alone. We quantified this effect by modelling responses based on Clyne’s models with upper and lower *EC*_50_ values for the native P2X2 receptor of 37 µM and 6.6 µM, and *n*_*H*_ = 2.4 as reported by Clyne *et al*.^12^. Figure 3c shows the predicted concentration-response curve when these two populations of P2X2 channels are equally represented in a mixed population, giving a predicted *n*_*H*_ of 1.4. Figure 3d shows the predicted change in *n*_*H*_ and *EC*_50_ as the proportions of the receptor subtypes vary. By contrast, both our data (Fig. 1c) and Clyne’s (their Fig. 3C) show that as *I*_*max*_ increases, there is no comparable change in *n*_*H*_; Fujiwara & Kubo^13^ report the same finding. There is also no tendency for the *EC*_50_s to plateau at the extreme ends of the *I*_*max*_ range as might be expected when one or other of the two subtypes is predominant. Figure 3e shows Clyne’s data with their proposed hyperbolic model (from which their *EC*_50_ values are derived) superimposed, yet there is little indication that the data follow the asymptotes of their model. Similarly, Fig. 3f shows data Clyne *et al*. obtained from a mutant receptor overlain on their model. Both of these datasets therefore cast doubt on the accuracy of their estimates of the *EC*_50_s for the distinct receptor subtypes. Indeed, it is likely that the upper and lower *EC*_50_ values they report were obtained because they constrained the lower *EC*_50_ values to be “no smaller than the lowest measured *y (i.e., EC*_50_) value” of 6.6 µM for the native receptors (Fig. 3e) and 4.8 µM for the mutant receptors (Fig. 3f).

Clyne’s second model proposes that the proportion of channels in a dimeric rather than monomeric conformation increases with higher expression levels. It does not require the presence of an endogenous binding protein and instead is predicted by mass action equilibrium in which channels may associate to form dimers (Fig. 4). If dimeric receptors had lower *EC*_50_ values than monomers, it is suggested that as channel expression levels increase, the observed *EC*_50_ would fall. However, as explained above, such a model with functionally discrete receptor subtypes implies that *n*_*H*_ would vary with the relative proportions of dimers and monomers, but this is not observed by Clyne *et al*.^12^. Furthermore, the relationship between *I*_*max*_ and *pEC*_50_ would still be expected to plateau since, at extreme low and high expression levels, channels would be in predominantly monomeric or dimeric forms. *I*_*max*_ values measured in our study range from 0.05–12.1 µA, implying a greater than 200-fold change in expression levels. Figure 4b indicates that, over a 100-fold range of receptor expression that would maximise the shift from monomeric to dimeric forms (i.e., 0.1 < A_TOT_·*K*_*A*_ < 10), the proportions of the two receptor populations reaching the asymptotes would be minimal, and therefore, *EC*_50_ values may not necessarily plateau, consistent with both our observations and those of Clyne *et al*.^12^. However, changes in *n*_*H*_ would be expected as described above, particularly in our model system where *n*_*H*_ is higher than that observed by Clyne *et al*.^12^. Indeed, at higher *n*_*H*_ values, the response observed in the presence of a population of two receptor subtypes would likely manifest as a biphasic concentration-response, which we did not observe. A further complication is that in a dynamic system, as envisaged in Fig. 4a, a ligand with a different affinity for monomers and dimers will tend to increase the proportion of receptors in the high affinity state (dimers in this case) as occupancy increases. Nevertheless, even under these circumstances, a biphasic or shallow concentration-response curve would be expected, even if the inflection point in the curve were less well defined or easily predicted. In summary, it is unlikely that the models proposed by Clyne *et al*. (2003) can account for either our or their observations.

**Figure 5:**
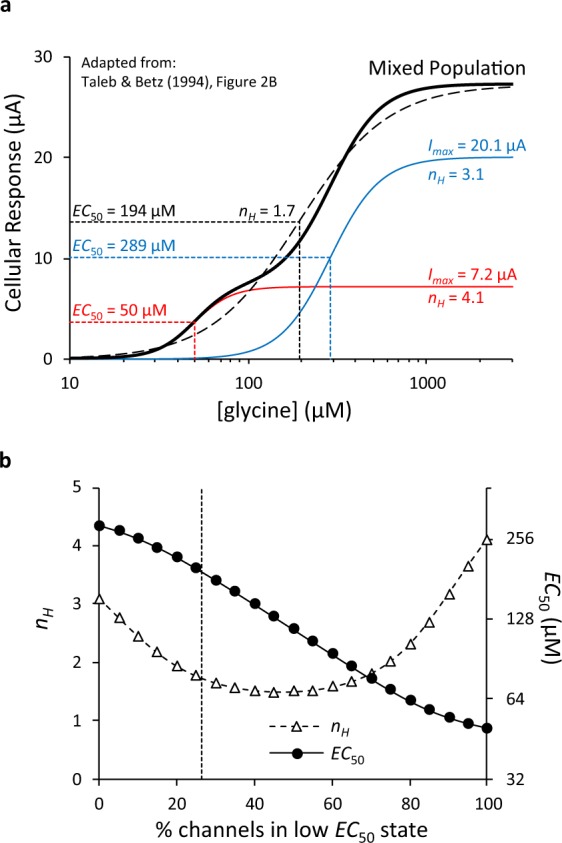
A representation and review of data from Taleb and Betz (1994). (**a**) Representation of a mixed population of glycine receptors with differing *EC*_50_ and *n*_*H*_ values derived from Fig. 2B of Taleb & Betz^15^. The mixed population (thick, black line) is the sum of two distinct populations (Red: *I*_*max*_ = 7.2 µA, *EC*_50_ = 50 µM, *n*_*H*_ = 4.1; Blue: *I*_*max*_ = 20.1 µA, *EC*_50_ = 289 µM, *n*_*H*_ = 3.1). At maximal glycine concentrations, the low *EC*_50_ population conducts approximately 25% of the total current and the high *EC*_50_ population conducts approximately 75% of the total current. The *EC*_50_ values are sufficiently different that the mixed population has a biphasic pattern. A monophasic concentration-response model derived from such a scenario would resemble the dashed line and thus have an intermediate *EC*_50_ of 194 µM, and a lower *n*_*H*_ of 1.7. (**b**) Predicted changes in overall *EC*_50_ and *n*_*H*_ as the proportions of channels in low and high *EC*_50_ states vary. The dashed line indicates the scenario depicted in Panel (**a**).

The emergence of a distinct high affinity subpopulation of receptors at high expression levels has been reported by Taleb & Betz^15^. They proposed that lateral allosteric interactions between glycine receptors may be responsible for the formation of a supra-molecular structure with altered gating requirements, and that receptor over-expression would lead to the formation of a higher proportion of these channel species as described in Fig. 4. The biphasic concentration-response curve shown in Fig. 2B of Taleb & Betz^15^ represents the summed responses from two presumed sub-populations of receptors with low and high *EC*_50_ values (*EC*_50.1_ = 50 µM, *I*_*max*.1_ = 7.2 µA, *n*_*H*.1_ = 4.1; *EC*_50.2_ = 289 µM, *I*_*max*.2_ = 20.1 µA, *n*_*H*.2_ = 3.1; shown in our Fig. 5a). These observations are consistent with the prediction that *n*_*H*_ will vary as the proportion of high and low *EC*_50_ receptors varies (Fig. 5b). As we found that *n*_*H*_ did not vary, it is not clear that the underlying mechanism driving the results of Taleb & Betz^15^ is the same as that causing the *I*_*max*_-dependent changes in *pEC*_50_ that we observe.

We therefore propose an alternative hypothesis that could account for our observations. Our explanation lies in considering the fate of a ligand immediately after it dissociates from its binding site. Although our experimental data do not directly measure ligand binding, they do provide a measurement of direct functional consequences, with increased channel opening at higher expression levels indicating increased binding site occupancy by 5-HT. The proposed mechanism addresses a key question: why, for a given ligand concentration, does proportional occupancy increase with receptor density, or more simply, why does ligand affinity gradually increase with increased receptor density?

The basic Langmuir model assumes that from one moment to the next, an unbound ligand molecule in solution may either remain in solution or become bound to a receptor. The rate at which ligand molecules bind to receptors is a function of an association rate constant, the concentration of the ligand in solution, and the concentration of unbound receptor. Similarly, a ligand bound to a receptor may either remain bound or dissociate and enter solution at a rate that is a function of a dissociation rate constant and the concentration of ligand bound receptors^2^. If on dissociating from one receptor a ligand is sufficiently close to another, it may bind directly to that other receptor. In effect, the ligand ‘hops’ from one receptor directly to another and unbinding from the first receptor, determined by the dissociation rate constant, is therefore not associated with a reduction in overall receptor occupancy. Receptors moving freely in a membrane will randomly come into closer proximity with a probability that increases as receptor density increases. Although the kinetics of individual ligand-receptor interactions would remain unchanged, more ‘ligand-hopping’ because of increased receptor densities would manifest as an overall (macroscopic) reduction in the dissociation rate constant, and therefore an apparent increase in ligand affinity. This would occur alongside an increase in the microscopic association rate constant since the on-rate, driven as expected by free ligand concentration and concentration of empty binding sites would be supplemented by the binding caused by ligand-hopping from adjacent receptors.

The probability that receptors will randomly collide with each other will depend upon the surface density of those receptors and their rate of lateral movement in the membrane. Assuming a single channel conductance of 0.4 pS^6^, and a near maximal open channel probability^16^ our maximal observed current (12.1 µA) at −60 mV suggests the presence of roughly 5 × 10^8^ channels which, for a 1 mm diameter oocyte, indicates a density of 160 channels/µm^2^. The cross-sectional area of a 5-HT_3_ receptor, approximately 0.00005 µm^2^, indicates a maximal packed density of 20,000 channels/µm^2^, which is clearly biologically impossible for a whole cell and overall receptor density will therefore be lower. Nevertheless, these figures suggest that if the number of channels we measured were randomly distributed on the cell surface they may not be sufficiently crowded to promote ligand-hopping. However, high density expression can occur in discrete regions of cell surfaces, for example, the nicotinic receptor at motor end plates of *Torpedo marmorata*^17^, and discrete regions of receptor clustering have been reported in *Xenopus* oocytes^18,19^. Within such regions, the density of receptors and their proximity could be high enough to facilitate ligand-hopping. Assuming that all 5-HT_3_ receptors were found within these clusters and that receptor density within the clusters was directly related to overall expression, ligand-hopping could provide a simple mechanistic explanation for the reduced *EC*_50_ associated with increased channel density. There would also be no transition shift in the Hill coefficient associated with functionally discrete subpopulations of receptors, and no need for alternative channel properties as a result of oligomer formation.

Others have suggested that the apparent dissociation rate is reduced at higher receptor densities. Erickson *et al*.^20^ claimed that when receptors cluster the “reverse rate constant is reduced because a ligand that dissociates from one receptor has a finite probability of binding to another before escaping from the vicinity of the cell”, and Gopalakrishnan *et al*.^21^ quantified a reduction in dissociation rate due to clustering. Elsewhere, Caré & Soula^22^ also incorporated this phenomenon into their model, but they concluded that ‘apparent’ affinity reduced with receptor clustering owing to a reduction in the association rate that overcame the reduction in dissociation rate. Such a conclusion is not consistent with our experimental findings, but clearly illustrates the challenges and complexity of predicting how clustering can affect observed pharmacological properties.

**Figure 6:**
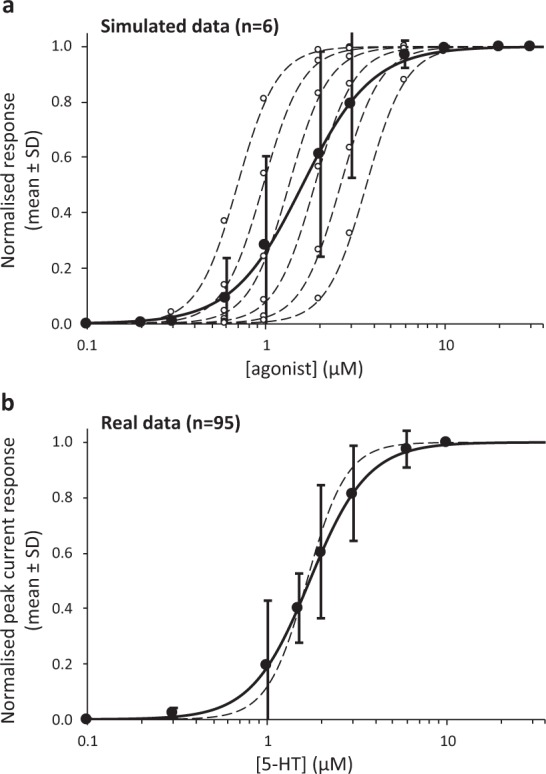
Effect of pooling concentration-response data on estimates of the Hill coefficient (*n*_*H*_). (**a**) Six simulated data sets are shown (- - - -) each with 11 data points (○) ranging from 0.1 to 30 µM, a normalised maximum of 1, and a Hill coefficient of 3.9. The *pEC*_50_ values differ and are: 6.20, 6.06, 5.92, 5.78, 5.64, 5.50. The mean ± SD of the six data points at each concentration are shown. A line of best fit (*OLS*) through the means is a 3PL model (——) with the maximum constrained to 1. The estimated *pEC*_50_ = 5.85 and *n*_*H*_ = 2.26. It is evident that the estimated *n*_*H*_ is biased down compared to the individual concentration-response data, and that there is substantial variance in the central part of the mean concentration-response curve. This pattern is expected when data are normalised and the *pEC*_50_s vary from one dataset to another. The extent of the bias is difficult to predict, but depends on the variance in the *pEC*_50_ values, the number of datasets, and the number and location of the data points. (**b**) Data from each of the 95 oocytes in our study were normalised to the response induced by 10 µM 5-HT. The mean ± SD for each 5-HT concentration was calculated from these normalised values. A 3PL model (——) was fitted (*OLS*) to the mean values with the maximum constrained to 1. The estimated *pEC*_50_ = 5.77 and *n*_*H*_ = 2.69. The dashed line (- - - -) shows the 3PL model defined by estimates shown in Table 1 (*pEC*_50_ = 5.78; *n*_*H*_ = 3.94).

A common analytical strategy for accommodating between-subject variance in current magnitude is to normalise data to a presumed maximal value. In addition, investigators may average concentration-response data and then fit a model curve to mean values. For example, Fig. 2A of Solt *et al*. (2007) shows a 5-HT-induced concentration-response “normalized to the peak current evoked by 100 µM 5-HT in the same cell”^23^. Normalisation and a ‘naïve-pooled’^24^ approach to analysis can generate misleading results by obscuring possible correlations between *I*_*max*_ and other parameters, and by reducing the accuracy of the estimates of those parameters, in particular the Hill coefficient. To illustrate this we performed a simple simulation. Figure 6a shows six simulated concentration-response curves with responses from 11 different concentrations (from 0.1 to 30 µM). Each curve has the same normalised maximal response (*I*_*max*_ = 1.0) and Hill coefficient (*n*_*H*_ = 3.9). The only difference is in the *pEC*_50_ values: 6.20, 6.06, 5.92, 5.78, 5.64, 5.50 (these values were selected to reflect those in Table 1). The mean ± SD of these six datasets for the 11 concentrations are shown, together with a three parameter logistic (3PL, Eq. (1)) model fitted to the means using ordinary least squares, with the maximum constrained to 1. The figure illustrates how these ‘ideal’ simulations generate substantial variance in the middle of the ‘average’ concentration-response curve, which is shallower than all the individual curves. In this simulation, the parameter estimates were: *pEC*_50_ = 5.85 ± 0.007 and *n*_*H*_ = 2.26 ± 0.07 (mean ± SE, *R*^2^ = 99.93%). To further illustrate this effect we re-analysed our real data (n = 95 oocytes) using the normalisation and naïve-pooled approach. For each oocyte, the current was normalised to the response induced by 10 µM 5-HT. Mean ± SD was calculated for each concentration of 5-HT and a 3PL model fitted (using *OLS*) with *I*_*max*_ constrained to 1. The data and model are shown in Fig. 6b. In this reanalysis, the parameter estimates were: *pEC*_50_ = 5.77 ± 0.003 and *n*_*H*_ = 2.69 ± 0.06 (mean ± SE; *R*^2^ = 99.96%). Figure 6b also shows the curve defined by the more accurate *pEC*_50_ and *n*_*H*_ values from Table 1. It is particularly striking that the estimate of *n*_*H*_ is lower than that obtained from every oocyte in Table 1 (minimum *n*_*H*_ = 2.74). These analyses show that normalisation and naïve-pooling obscure potential correlations and generate biased estimates of the Hill coefficient. Not only may estimates be inaccurate, but *pEC*_50_ and *n*_*H*_ may also have a precision that is unwarranted (note the high *R*^2^ values and low standard error estimates) and that fails to reflect the normal variability in real data. Finally, since Hill coefficients may be interpreted mechanistically, particularly in ion channels, it is important to obtain unbiased estimates of these values to avoid inaccurate interpretation of ion channel function and pharmacology.

In conclusion, we provide evidence that at ligand-gated ion channels, the *EC*_50_ of an agonist decreases as *I*_*max*_ increases. Since *I*_*max*_ is proportional to the number of receptors on the cell-surface and ligand binding is directly associated with channel opening, our results indicate that increased receptor density is associated with increased proportional receptor occupancy for a given agonist concentration. Although our data are unable to confirm any specific mechanistic explanation, we show that they are inconsistent with some previously offered mechanisms. We propose an alternative mechanistic hypothesis, that increased expression levels may result in closer proximity between receptors in localised areas of high receptor density, thereby facilitating a mechanism of ‘ligand-hopping’ between receptors. This effect would incrementally increase as receptor density increases and is not dependent on functionally distinct populations of receptors. Importantly, this relationship between *I*_*max*_ and *EC*_50_ could adversely affect the accuracy and precision of measured pharmacological data when absolute current amplitudes are concealed by normalisation and naïve pooling of data. By contrast, explicit recognition of these issues and their accommodation into the design and analysis of experiments promotes the generation of more accurate quantitative pharmacological data.

## Materials and Methods

### Constructs

Human 5-HT3A (accession number: P46098) subunit cDNA was kindly provided by J. Peters (Dundee University, UK) and was cloned into pGEMHE for oocyte expression^25^.

### Oocyte maintenance and receptor expression

Oocytes from *Xenopus laevis* were purchased from EcoCyte Bioscience (Castrop-Rauxel, Germany) and stored at 16 °C in ND96 (96 mM NaCl, 2 mM KCl, 1 mM MgCl_2_, 5 mM HEPES, pH 7.5). 5-HT3A subunit cRNA was *in vitro* transcribed from linearised plasmid cDNA template using the mMESSAGE mMACHINE T7 Ultra Transcription Kit (Ambion, Austin, Texas, USA). Stage V and VI oocytes were injected with 50 nl of 3.5–500 ng·µl^−1^ cRNA (0.175–25 ng injected), and currents were recorded at varying times up to 7 days post-injection^3^. Monomeric 5-HT_3_A receptors were selected as a model system since they do not show any constitutive activity in the absence of 5-HT^16,26^.

### Electrophysiology

Using two electrode voltage clamp (TEVC), *Xenopus* oocytes were clamped using an OC-725 amplifier (Warner Instruments, Connecticut, USA), NI USB-6341 X Series DAQ Device (National Instruments, Berkshire, UK) and the Strathclyde Electrophysiology Software Package v4.7.3 (http://spider.science.strath.ac.uk/sipbs/software_ses.htm; University of Strathclyde, UK). Micro-electrodes were fabricated from borosilicate glass (GC120TF-10, Harvard Apparatus, Edenbridge, Kent, UK) using a two stage horizontal pull (P-1000, Sutter Instrument Company, California, USA) and filled with 3 M KCl. Pipette resistances ranged from 0.8–2.0 MΩ. Oocytes were placed in a perfusion chamber made from 2 mm wide × 30 mm long silicon tubing that was cut in half lengthways (total volume ~ 0.1 ml), and were perfused with ND96 at a rate of 12 ml·min^−1^. Agonist application was via a simple gravity fed system calibrated to run at the same rate with a 2 min wash used between applications^3^. In studies such as this, TEVC is advantageous as there is only a small series resistance error due to the voltage drop resulting from current flow to the reference/ground electrode across the extracellular fluid, ensuring comparability of responses in oocytes with different peak currents. Cytosolic series resistance (R_c_) caused by the large volume of oocytes is unlikely to be an issue for our experiments. For a R_c_ of 0.2 kΩ in *Xenopus* oocytes^27^, a maximal peak current of 10 µA would lead to a deviation of 2 mV, meaning that a command voltage of −60 mV would result in a membrane voltage of −58 mV. Baumgartner *et al*. concluded that an oocyte cannot be considered isopotential on time scales of 300 µs or less, or when the total current is larger than ~20 µA^28^, and since neither of these conditions were exceeded in our experiments, it suggests that the bias introduced by cytosolic series resistance in our system was minimal. Leak currents were recorded immediately prior to 5-HT application and subtracted from subsequent 5-HT evoked responses.

### Data and statistical analysis

Peak currents evoked by different concentrations of 5-HT in individual oocytes were modelled using the following three parameter logistic (3PL) equation:

1 $${I}_{PRED}=\frac{{I}_{max}}{1+{({10}^{-pE{C}_{50}}/[A])}^{{n}_{H}}}$$

where: *I*_*PRED*_ = the predicted peak current (µA), and [*A*] is the concentration of 5-HT (mol·L^-1^). *I*_*max*_ = peak current evoked by a maximal concentration of 5-HT; *pEC*_50_ = negative logarithm of the concentration of 5-HT which gives a response equal to *I*_*max*_/2; *n*_*H*_ = Hill coefficient, which is a measure of the ‘steepness’ of the observed agonist-response relationship. The 3PL model assumes that when [5-HT] = 0, there is no response, i.e., *I*_*PRED*_ = 0. Monomeric 5-HT_3_A receptors were used since they have been reported to show no constitutive activity^16,26^, as our data confirmed. The mid-point concentration was modelled as the *pEC*_50_ since random error around this value is distributed log-normally^29^.

When there is no response, the variance is also low, and assumed to be zero. As current amplitude increases, the variance in the response also increases, in common with much biological concentration-response data. Model fitting with ordinary least squares (*OLS*) overlooks this and can result in biased estimates of parameters. To avoid this, we modelled our data using a maximum likelihood approach, enabling the relationship between size and variability of response to be accommodated and quantified.

Residual error was modelled as follows:

2 $$RU{V}_{j}={\alpha }^{2}{I}_{PREDj}^{\gamma }$$

where: *RUV*_*j*_ = residual unexplained variance for *j*^*th*^ recording; *α* is a variance parameter modified by the predicted response (*I*_*PREDj*_) for *j*^*th*^ recording; *γ* modifies the relationship between *I*_*PREDj*_ and *RUV*_*j*_. When *γ* = 0, residual error is homoscedastic; when *γ* = 2, the coefficient of variation of the residual error is constant.

The objective function used to generate best fit models was the extended least squares (*ELS*)^30–32^, which incorporates estimates of the residual unexplained variance.

*ELS* was calculated as follows:

3 $$ELS=\mathop{\sum }\limits_{j=1}^{n}(\frac{{({I}_{OBSj}-{I}_{PREDj})}^{2}}{RU{V}_{j}}+ln(RU{V}_{j}))$$

where: *n* = the number of recordings per oocyte; *I*_*OBSj*_ = the observed peak current for the *j*^th^ recording; *I*_*PREDj*_ = the predicted peak current for the *j*^th^ recording.

Each oocyte therefore generated five parameters: *I*_*max*_; *pEC*_50_; *n*_*H*_; *α*; *γ*). Model fitting was performed using the Solver function in Microsoft Excel and NONMEM 7.3.0 (Icon PLC, Dublin, Ireland) with Wings for NONMEM (distributed under a GNU General Public licence). Fitted models were constrained such that 0 ≤ *γ* ≤ 2. Estimates of the modelled parameters and other metadata for each oocyte are in Supplementary Table 1.

Data were also analysed to investigate current run-down during the experiment by modelling *I*_*max*_ as a linear function of time from the start of the experiment. Each concentration of 5-HT took approximately 1 minute to complete. This analysis showed that run-down, as a percentage of the initial *I*_*max*_, was low (mean ± sd = −0.007 ± 1.937% min^−1^). Consequently, results are reported from the analysis without run-down.

Owing to the practicalities of the experimental setup, the investigators could not be blinded to the dose of RNA injected into oocytes or to the length of time of incubation between injection and measurement. Similarly, the investigator was not blinded to the concentrations of 5-HT used in each experiment.

## Supplementary Information

Supplementary Information

## Data Availability

The datasets generated and analysed during the current study are available on request.
