# Supplementary material for: Evidence for an effect of receptor density on ligand occupancy and agonist EC50
Source: Sci Rep. 2019 Dec 13;9:19111. doi: 10.1038/s41598-019-55361-x (PMC6910986; doi:10.1038/s41598-019-55361-x)
Supplement: Supplementary file 1 — Supplementary Information [file 41598_2019_55361_MOESM1_ESM.pdf]

| Oocyte ID# | Oocyte Code | RNA injection date/time | RNA injection conc (µg/µl) | RNA injection dose (ng) | Electrophysiology Experiment start time | Number of measurements per oocyte | $I_{max}$ | $pEC_{50}$ | $n_H$ | alpha ($\alpha$) | gamma ($\gamma$) | *ELS* | Comments |
|---|---|---|---|---|---|---|---|---|---|---|---|---|---|
| 1 | 42537c1 | 14/06/2016 16:00 | 0.5 | 25 | 16/06/2016 10:01 | 15 | 6.4036394 | 5.7552687 | 3.6463815 | 0.0830413 | 0.5724845 | -58.90496 | |
| 2 | 42537c2 | 14/06/2016 16:00 | 0.5 | 25 | 16/06/2016 10:21 | 14 | 6.5904024 | 5.729241 | 3.5801981 | 0.0710765 | 0.8109716 | -60.90685 | |
| 3 | 42537c3 | 14/06/2016 16:00 | 0.5 | 25 | 16/06/2016 10:41 | 14 | 7.8801436 | 5.797709 | 3.8213889 | 0.2492967 | 0.7778165 | -22.5827 | |
| 4 | 42537c4 | 14/06/2016 16:00 | 0.5 | 25 | 16/06/2016 11:01 | 14 | 6.5394076 | 5.791984 | 3.7384221 | 0.1040882 | 0.6738948 | -49.03525 | |
| 5 | 42537c5 | 14/06/2016 16:00 | 0.5 | 25 | 16/06/2016 11:21 | 14 | 6.5502006 | 5.8225126 | 3.6436289 | 0.111554 | 0.8464833 | -45.3967 | |
| 6 | 42538c1 | 14/06/2016 16:00 | 0.5 | 25 | 17/06/2016 10:01 | 14 | 7.7759769 | 5.7038462 | 3.8605259 | 0.147716 | 0.7627217 | -42.49362 | |
| 7 | 42538c2 | 14/06/2016 16:00 | 0.5 | 25 | 17/06/2016 10:21 | 14 | 7.287489 | 5.6899252 | 3.7811307 | 0.075364 | 0.5971843 | -61.3476 | |
| 8 | 42538c3 | 14/06/2016 16:00 | 0.5 | 25 | 17/06/2016 10:41 | 14 | 8.2282412 | 5.8021157 | 3.8693963 | 0.0457028 | 0.5461037 | -71.28684 | |
| 9 | 42538c4 | 14/06/2016 16:00 | 0.5 | 25 | 17/06/2016 11:01 | 14 | 7.2341013 | 5.7059162 | 3.8025813 | 0.064788 | 0.2581005 | -63.7402 | |
| 10 | 42538c5 | 14/06/2016 16:00 | 0.5 | 25 | 17/06/2016 11:21 | 14 | 7.2570159 | 5.7277671 | 3.8314603 | 0.0797543 | 0.6571571 | -57.33033 | |
| 11 | 42538c6 | 14/06/2016 16:00 | 0.5 | 25 | 17/06/2016 11:41 | 14 | 7.8859276 | 5.7889356 | 3.7422348 | 0.0347845 | 0.4004182 | -79.39904 | |
| 12 | 42538c7 | 14/06/2016 16:00 | 0.5 | 25 | 17/06/2016 12:01 | 14 | 7.4940137 | 5.77055 | 3.8505967 | 0.0573984 | 1.0701374 | -66.78389 | |
| 13 | 42538c8 | 14/06/2016 16:00 | 0.5 | 25 | 17/06/2016 12:21 | 15 | 7.7913584 | 5.8148948 | 4.2250002 | 0.2185956 | 0.7253056 | -30.74229 | |
| 14 | 42538c9 | 14/06/2016 16:00 | 0.5 | 25 | 17/06/2016 14:01 | 15 | 7.4802079 | 5.8244045 | 3.6437929 | 0.0914049 | 0.9104113 | -52.14119 | |
| 15 | 42538c10 | 14/06/2016 16:00 | 0.5 | 25 | 17/06/2016 14:21 | 14 | 7.948145 | 5.8733009 | 3.7047257 | 0.083878 | 0.7273227 | -51.30781 | |
| 16 | 42642c1 | 27/09/2016 15:40 | 0.095 | 4.75 | 29/09/2016 10:01 | 7 | 7.5166717 | 5.9315394 | 4.4894511 | 0.040487 | 2 | -34.16669 | $\gamma$ constrained to 2 |
| 17 | 42642c2 | 27/09/2016 15:45 | 0.287 | 14.35 | 29/09/2016 10:11 | 7 | 8.6256968 | 6.0011184 | 3.8434234 | 0.0373312 | 2 | -28.08762 | $\gamma$ constrained to 2 |
| 18 | 42642c3 | 27/09/2016 15:45 | 0.287 | 14.35 | 29/09/2016 10:24 | 5 | 8.3315558 | 5.9981479 | 4.0104897 | 0.0200931 | 2 | -14.4256 | $\gamma$ constrained to 2 |
| 19 | 42642c4 | 27/09/2016 15:45 | 0.287 | 14.35 | 29/09/2016 10:38 | 7 | 8.2738087 | 5.9874688 | 3.4868632 | 0.0214533 | 2 | -35.19664 | $\gamma$ constrained to 2 |
| 20 | 42642c5 | 27/09/2016 15:40 | 0.095 | 4.75 | 29/09/2016 10:50 | 7 | 6.6074247 | 5.8285884 | 3.9474 | 0.1137881 | 0.838609 | -23.02213 | |
| 21 | 42642c6 | 27/09/2016 15:40 | 0.095 | 4.75 | 29/09/2016 11:50 | 7 | 8.9849402 | 5.8633407 | 3.6744709 | 0.0612865 | 1.226318 | -27.14176 | |
| 22 | 42641c1 | 27/09/2016 15:45 | 0.287 | 14.35 | 28/09/2016 10:15 | 10 | 5.3981721 | 5.7277339 | 3.8293932 | 0.1749042 | 1.0589758 | -22.35079 | |
| 23 | 42641c2 | 27/09/2016 15:40 | 0.095 | 4.75 | 28/09/2016 13:00 | 8 | 4.8303795 | 5.7363616 | 4.7444884 | 0.2929848 | 1.3154948 | -17.89105 | |
| 24 | 42641c3 | 27/09/2016 15:40 | 0.095 | 4.75 | 28/09/2016 13:29 | 6 | 6.0685534 | 5.7620067 | 3.9295556 | 0.0412357 | 2 | -16.2962 | $\gamma$ constrained to 2 |
| 25 | 42641c4 | 27/09/2016 15:45 | 0.287 | 14.35 | 28/09/2016 13:40 | 7 | 5.9577646 | 5.7944492 | 3.9151293 | 0.0364535 | 0.5657096 | -39.82879 | |
| 26 | 42641c5 | 27/09/2016 15:40 | 0.095 | 4.75 | 28/09/2016 14:10 | 8 | 5.6319641 | 5.7772127 | 3.8194192 | 0.041397 | 2 | -42.05214 | $\gamma$ constrained to 2 |
| 27 | 42641c7 | 27/09/2016 15:40 | 0.095 | 4.75 | 28/09/2016 14:25 | 7 | 5.3435187 | 5.7921366 | 3.8932863 | 0.042376 | 0.7652876 | -38.45284 | |
| 28 | 42641c8 | 27/09/2016 15:45 | 0.287 | 14.35 | 28/09/2016 14:36 | 7 | 6.8866156 | 5.7796054 | 4.3552784 | 0.0115223 | 0.8534067 | -56.76486 | |
| 29 | 42641c9 | 27/09/2016 15:40 | 0.095 | 4.75 | 28/09/2016 14:50 | 7 | 5.13484 | 5.7286882 | 3.6660611 | 0.0570555 | 0.9944589 | -35.51214 | |
| 30 | 42641c10 | 27/09/2016 15:40 | 0.095 | 4.75 | 28/09/2016 15:43 | 7 | 4.8322007 | 5.7292112 | 3.9205218 | 0.1087003 | 0.66532 | -26.53293 | |
| 31 | 42643c2 | 27/09/2016 15:40 | 0.095 | 4.75 | 30/09/2016 12:28 | 9 | 12.110336 | 6.0595821 | 3.9236265 | 0.2623771 | 1.5961397 | -0.39072 | |
| 32 | 42643c3 | 27/09/2016 15:40 | 0.095 | 4.75 | 30/09/2016 12:50 | 7 | 5.9832941 | 5.8076646 | 3.4250202 | 0.042384 | 1.2532381 | -36.08994 | |
| 33 | 42643c4 | 27/09/2016 15:45 | 0.287 | 14.35 | 30/09/2016 13:04 | 7 | 8.9845161 | 5.979136 | 4.3376756 | 0.0422189 | 0.8190879 | -33.78733 | |
| 34 | 42643c5 | 27/09/2016 15:40 | 0.095 | 4.75 | 30/09/2016 13:32 | 7 | 9.1879858 | 6.0240306 | 3.9685832 | 0.0125395 | 2 | -42.39273 | $\gamma$ constrained to 2 |
| 35 | 42643c6 | 27/09/2016 15:45 | 0.287 | 14.35 | 30/09/2016 13:46 | 7 | 9.9706182 | 6.1164219 | 3.9041631 | 0.0198982 | 2 | -32.19982 | $\gamma$ constrained to 2 |
| 36 | 42643c7 | 27/09/2016 15:40 | 0.095 | 4.75 | 30/09/2016 14:04 | 7 | 9.927546 | 6.0160669 | 3.3177023 | 0.0104638 | 2 | -41.27162 | $\gamma$ constrained to 2 |
| 37 | 42643c8 | 27/09/2016 15:45 | 0.287 | 14.35 | 30/09/2016 14:34 | 7 | 10.095817 | 6.0519091 | 5.3401473 | 0.1431535 | 0.8891349 | -16.57992 | |
| 38 | 42646c1 | 27/09/2016 15:40 | 0.095 | 4.75 | 03/10/2016 09:34 | 8 | 9.7731034 | 6.0807268 | 4.5210511 | 0.107094 | 2 | -11.17754 | $\gamma$ constrained to 2 |
| 39 | 42646c2 | 27/09/2016 15:45 | 0.287 | 14.35 | 03/10/2016 09:53 | 9 | 10.030095 | 6.1551424 | 3.5435229 | 0.103699 | 1.0778043 | -20.71363 | |
| 40 | 42646c3 | 27/09/2016 15:45 | 0.287 | 14.35 | 03/10/2016 10:19 | 8 | 9.4081875 | 6.1564706 | 4.1662901 | 0.1094076 | 1.4671336 | -18.48626 | data shown in Fig 1B |
| 41 | 42646c4 | 27/09/2016 15:40 | 0.095 | 4.75 | 03/10/2016 10:35 | 8 | 11.040834 | 6.0385972 | 4.9822414 | 0.0399905 | 1.6758214 | -39.98268 | |
| 42 | 42646c5 | 27/09/2016 15:45 | 0.287 | 14.35 | 03/10/2016 10:53 | 8 | 9.5563948 | 6.0979592 | 4.0117321 | 0.032228 | 2 | -28.24955 | $\gamma$ constrained to 2 |
| 43 | 42646c6 | 27/09/2016 15:40 | 0.095 | 4.75 | 03/10/2016 11:15 | 8 | 11.886465 | 6.057187 | 4.4035157 | 0.0552453 | 1.31291 | -31.72779 | |
| 44 | 42647c1 | 27/09/2016 15:45 | 0.287 | 14.35 | 04/10/2016 09:46 | 7 | 9.1125781 | 6.0959125 | 4.7633585 | 0.0154472 | 2 | -40.91033 | $\gamma$ constrained to 2 |
| 45 | 42647c2 | 27/09/2016 15:40 | 0.095 | 4.75 | 04/10/2016 10:15 | 7 | 9.4445988 | 6.0533377 | 5.8947266 | 0.0848374 | 2 | -22.60875 | $\gamma$ constrained to 2 |

| Oocyte ID# | Oocyte Code | RNA injection date/time | RNA injection conc (µg/µl) | RNA injection dose (ng) | Electrophysiology Experiment start time | Number of measurements per oocyte | $I_{max}$ | $pEC_{50}$ | $n_H$ | alpha ($\alpha$) | gamma ($\gamma$) | *ELS* | Comments |
|---|---|---|---|---|---|---|---|---|---|---|---|---|---|
| 46 | 42647c3 | 27/09/2016 15:40 | 0.095 | 4.75 | 04/10/2016 10:15 | 7 | 9.3294345 | 6.0864123 | 4.8131517 | 0.1576897 | 1.0188077 | -13.59779 | |
| 47 | 42647c4 | 27/09/2016 15:45 | 0.287 | 14.35 | 04/10/2016 10:38 | 8 | 8.5869891 | 6.130822 | 5.5739143 | 0.0428349 | 2 | -30.66483 | $\gamma$ constrained to 2 |
| 48 | 42648c1 | 05/10/2016 10:20 | 0.287 | 14.35 | 05/10/2016 15:12 | 1 | | | | | | | no response to 5-HT |
| 49 | 42648c2 | 05/10/2016 10:20 | 0.287 | 14.35 | 05/10/2016 15:16 | 2 | | | | | | | no response to 5-HT |
| 50 | 42648c3 | 05/10/2016 10:00 | 0.0316 | 1.58 | 05/10/2016 15:24 | 1 | | | | | | | no response to 5-HT |
| 51 | 42648c4 | 05/10/2016 10:10 | 0.095 | 4.75 | 05/10/2016 15:27 | 6 | 0.1459642 | 5.439216 | 2.7429837 | 0.0433772 | 2 | -68.53119 | $\gamma$ constrained to 2; data shown in Fig 1B |
| 52 | 42648c5 | 05/10/2016 10:10 | 0.095 | 4.75 | 05/10/2016 15:30 | 1 | | | | | | | no response to 5-HT |
| 53 | 42648c6 | 05/10/2016 10:10 | 0.095 | 4.75 | 05/10/2016 15:42 | 6 | 0.127077 | 5.5190085 | 3.4160176 | 0.0026747 | 0 | -65.08721 | $\gamma$ constrained to 0 |
| 54 | 42648c7 | 05/10/2016 10:10 | 0.095 | 4.75 | 05/10/2016 15:51 | 6 | 0.0498304 | 5.4569092 | 4.1623103 | 0.0066931 | 0.8647853 | -77.33403 | |
| 55 | 42648c8 | 05/10/2016 10:10 | 0.095 | 4.75 | 05/10/2016 15:59 | 7 | 0.1426774 | 5.5530092 | 4.7811695 | 0.0290928 | 0.9436012 | -62.28549 | |
| 56 | 42648c9 | 05/10/2016 10:10 | 0.095 | 4.75 | 05/10/2016 17:16 | 7 | 0.685333 | 5.5488339 | 3.6216381 | 0.0232161 | 0.8933357 | -64.22904 | |
| 57 | 42648c10 | 05/10/2016 10:20 | 0.287 | 14.35 | 05/10/2016 17:21 | 7 | 0.0752155 | 5.5218236 | 3.7094126 | 0.0143319 | 0.816037 | -82.90886 | |
| 58 | 42648c11 | 05/10/2016 10:10 | 0.095 | 4.75 | 05/10/2016 17:34 | 7 | 0.1275492 | 5.5370548 | 4.4799293 | 0.007769 | 0.4416613 | -77.27507 | |
| 59 | 42649c1 | 05/10/2016 10:00 | 0.0316 | 1.58 | 06/10/2016 09:54 | 7 | 3.0463569 | 5.7346436 | 3.6835427 | 0.0430693 | 0.9819911 | -42.94078 | |
| 60 | 42649c2 | 05/10/2016 10:00 | 0.0316 | 1.58 | 06/10/2016 10:04 | 7 | 2.167319 | 5.6819953 | 3.7758247 | 0.0549648 | 1.1533331 | -45.03598 | |
| 61 | 42649c3 | 05/10/2016 10:10 | 0.095 | 4.75 | 06/10/2016 10:16 | 7 | 5.5073051 | 5.8156023 | 3.3984361 | 0.0108543 | 2 | -55.19647 | $\gamma$ constrained to 2 |
| 62 | 42649c4 | 05/10/2016 10:30 | 0.0105 | 0.525 | 06/10/2016 10:23 | 7 | 3.4713825 | 5.7326723 | 3.7957414 | 0.0509546 | 1.0542893 | -40.49814 | |
| 63 | 42649c5 | 05/10/2016 10:00 | 0.0316 | 1.58 | 06/10/2016 10:34 | 7 | 4.8912334 | 5.782975 | 3.5486971 | 0.0126665 | 1.2203251 | -55.7558 | |
| 64 | 42649c6 | 05/10/2016 10:00 | 0.0316 | 1.58 | 06/10/2016 10:42 | 7 | 3.3581531 | 5.6717818 | 3.6310889 | 0.0591647 | 1.0429893 | -39.41327 | |
| 65 | 42649c8 | 05/10/2016 10:00 | 0.0316 | 1.58 | 06/10/2016 10:52 | 7 | 7.2211666 | 5.7402648 | 3.3401251 | 0.0989529 | 2 | -22.85057 | $\gamma$ constrained to 2 |
| 66 | 42649c9 | 05/10/2016 10:00 | 0.0316 | 1.58 | 06/10/2016 11:00 | 7 | 3.604898 | 5.7282473 | 4.6378093 | 0.2200361 | 1.0787097 | -23.06739 | |
| 67 | 42649c10 | 05/10/2016 10:00 | 0.0316 | 1.58 | 06/10/2016 11:17 | 7 | 4.1896243 | 5.7408952 | 3.6840599 | 0.0506093 | 0.6821205 | -37.26667 | |
| 68 | 42649c11 | 05/10/2016 10:00 | 0.0316 | 1.58 | 06/10/2016 11:27 | 7 | 5.4368778 | 5.7581024 | 3.9461274 | 0.0789648 | 1.1729522 | -31.26732 | |
| 69 | 42649c12 | 05/10/2016 10:00 | 0.0316 | 1.58 | 06/10/2016 12:06 | 7 | 5.4243766 | 5.7682526 | 4.3500796 | 0.0956921 | 1.3411451 | -30.42931 | |
| 70 | 42649c13 | 05/10/2016 10:30 | 0.0105 | 0.525 | 06/10/2016 13:32 | 7 | 3.9035772 | 5.7334831 | 4.4883863 | 0.1550836 | 0 | -19.09298 | $\gamma$ constrained to 0 |
| 71 | 42649c14 | 05/10/2016 10:30 | 0.0105 | 0.525 | 06/10/2016 13:43 | 7 | 5.0656089 | 5.7217354 | 3.8404124 | 0.0505833 | 0.906082 | -37.74161 | |
| 72 | 42649c15 | 05/10/2016 10:20 | 0.287 | 14.35 | 06/10/2016 13:51 | 7 | 8.000332 | 5.8627686 | 3.6371743 | 0.0300895 | 0.6495523 | -39.89683 | |
| 73 | 42649c16 | 05/10/2016 10:30 | 0.0105 | 0.525 | 06/10/2016 14:00 | 7 | 4.1383456 | 5.751784 | 3.9078812 | 0.0178316 | 2 | -36.59458 | $\gamma$ constrained to 2 |
| 74 | 42649c17 | 05/10/2016 10:30 | 0.0105 | 0.525 | 06/10/2016 14:09 | 8 | 4.8067126 | 5.7599313 | 3.6072653 | 0.0384422 | 1.0532038 | -45.1389 | |
| 75 | 42649c18 | 05/10/2016 10:30 | 0.0105 | 0.525 | 06/10/2016 14:37 | 7 | 3.7405749 | 5.7697439 | 3.7021538 | 0.040181 | 1.2554103 | -42.91029 | |
| 76 | 42649c19 | 05/10/2016 10:10 | 0.095 | 4.75 | 06/10/2016 14:45 | 7 | 5.3887394 | 5.8163394 | 3.4811332 | 0.0352604 | 0.9555365 | -39.65335 | |
| 77 | 42649c21 | 05/10/2016 10:30 | 0.0105 | 0.525 | 06/10/2016 14:55 | 7 | 2.7980556 | 5.7391502 | 4.3515356 | 0.0717177 | 0.9585764 | -38.27542 | |
| 78 | 42649c22 | 05/10/2016 10:30 | 0.0105 | 0.525 | 06/10/2016 15:05 | 7 | 3.7037261 | 5.7448706 | 3.6718637 | 0.0405253 | 1.0830316 | -42.64583 | |
| 79 | 42649c23 | 05/10/2016 10:30 | 0.0105 | 0.525 | 06/10/2016 15:50 | 7 | 4.623691 | 5.747509 | 3.7686084 | 0.0338959 | 1.3955647 | -44.70636 | |
| 80 | 42649c24 | 05/10/2016 10:10 | 0.095 | 4.75 | 06/10/2016 15:57 | 7 | 3.2522937 | 5.739681 | 3.8786409 | 0.0123722 | 1.1647388 | -61.59934 | |
| 81 | 42649c26 | 05/10/2016 10:30 | 0.0105 | 0.525 | 06/10/2016 16:07 | 7 | 4.019645 | 5.7665003 | 3.992968 | 0.0062874 | 1.0484303 | -68.58782 | |
| 82 | 42649c27 | 05/10/2016 10:20 | 0.287 | 14.35 | 06/10/2016 16:16 | 8 | 7.4011151 | 5.7521834 | 3.6591149 | 0.230251 | 1.3853781 | -12.52269 | |
| 83 | 42650c1 | 06/10/2016 16:30 | 0.0035 | 0.175 | 07/10/2016 09:19 | 7 | 0.3443189 | 5.5575646 | 3.6154277 | 0.0142269 | 1.171174 | -82.12384 | |
| 84 | 42650c2 | 06/10/2016 16:45 | 0.0105 | 0.525 | 07/10/2016 09:27 | 7 | 0.8770038 | 5.5827066 | 3.7289328 | 0.0333533 | 0.9618248 | -58.43741 | |
| 85 | 42650c3 | 06/10/2016 16:45 | 0.0105 | 0.525 | 07/10/2016 09:34 | 7 | 0.9790654 | 5.5990753 | 4.0055288 | 0.0363065 | 1.11841 | -60.09791 | |
| 86 | 42650c4 | 06/10/2016 16:45 | 0.0105 | 0.525 | 07/10/2016 09:41 | 7 | 2.1246779 | 5.6252517 | 3.6602001 | 0.0299229 | 1.2137824 | -55.42767 | |
| 87 | 42650c5 | 06/10/2016 16:45 | 0.0105 | 0.525 | 07/10/2016 09:49 | 7 | 1.0385177 | 5.6097403 | 3.8817957 | 0.0182305 | 1.3016717 | -71.54162 | |
| 88 | 42650c6 | 06/10/2016 16:30 | 0.0035 | 0.175 | 07/10/2016 09:57 | 7 | 0.655623 | 5.5803262 | 3.7667522 | 0.0317639 | 0.7960198 | -57.84935 | |
| 89 | 42650c7 | 06/10/2016 16:30 | 0.0035 | 0.175 | 07/10/2016 10:04 | 7 | 0.4572032 | 5.5579525 | 3.6434244 | 0.0424089 | 1.0342112 | -61.43814 | |
| 90 | 42650c8 | 06/10/2016 16:45 | 0.0105 | 0.525 | 07/10/2016 10:10 | 7 | 1.6291148 | 5.6575439 | 3.6923715 | 0.0275864 | 1.4170139 | -60.4893 | |

| Oocyte ID# | Oocyte Code | RNA injection date/time | RNA injection conc (µg/µl) | RNA injection dose (ng) | Electrophysiology Experiment start time | Number of measurements per oocyte | $I_{max}$ | $pEC_{50}$ | $n_H$ | alpha ($\alpha$) | gamma ($\gamma$) | *ELS* | Comments |
|---|---|---|---|---|---|---|---|---|---|---|---|---|---|
| 91 | 42650c9 | 06/10/2016 16:45 | 0.0105 | 0.525 | 07/10/2016 10:18 | 7 | 1.8610461 | 5.6556993 | 3.630303 | 0.0186686 | 1.2406886 | -62.41948 | |
| 92 | 42650c10 | 06/10/2016 16:45 | 0.0105 | 0.525 | 07/10/2016 10:24 | 7 | 2.056106 | 5.6053995 | 4.0043731 | 0.0304635 | 1.4593398 | -61.0118 | |
| 93 | 42650c11 | 06/10/2016 16:45 | 0.0105 | 0.525 | 07/10/2016 10:31 | 7 | 1.002424 | 5.5759113 | 3.6667588 | 0.0329888 | 1.2802619 | -63.18639 | |
| 94 | 42650c12 | 06/10/2016 16:30 | 0.0035 | 0.175 | 07/10/2016 10:37 | 7 | 0.5738051 | 5.5746742 | 3.786813 | 0.0304138 | 1.01091 | -64.11513 | |
| 95 | 42650c13 | 06/10/2016 16:30 | 0.0035 | 0.175 | 07/10/2016 10:44 | 7 | 0.391043 | 5.5797375 | 3.8673924 | 0.0277579 | 1.1463879 | -71.68792 | |
| 96 | 42650c15 | 06/10/2016 16:30 | 0.0035 | 0.175 | 07/10/2016 10:53 | 7 | 0.5776141 | 5.5835782 | 3.7807785 | 0.036576 | 1.0157427 | -61.35302 | |
| 97 | 42650c16 | 06/10/2016 16:30 | 0.0035 | 0.175 | 07/10/2016 12:50 | 7 | 0.1563187 | 5.561691 | 4.6767275 | 0.1246865 | 1.1053248 | -61.33233 | |
| 98 | 42650c17 | 06/10/2016 16:30 | 0.0035 | 0.175 | 07/10/2016 12:56 | 7 | 0.5078711 | 5.5192087 | 3.0182521 | 0.0092764 | 0 | -58.52394 | $\gamma$ constrained to 0 |
| 99 | 42650c18 | 06/10/2016 16:30 | 0.0035 | 0.175 | 07/10/2016 13:03 | 7 | 0.410184 | 5.5664063 | 3.9299608 | 0.054118 | 0.9715077 | -58.3021 | |
